# Structural, Evolutionary, and Functional Analysis of the Class III Peroxidase Gene Family in Chinese Pear (*Pyrus bretschneideri*)

**DOI:** 10.3389/fpls.2016.01874

**Published:** 2016-12-09

**Authors:** Yunpeng Cao, Yahui Han, Dandan Meng, Dahui Li, Qing Jin, Yi Lin, Yongping Cai

**Affiliations:** ^1^School of Life Sciences, Anhui Agricultural UniversityHefei, China; ^2^State Key Laboratory of Tea Plant Biology and Utilization, Anhui Agricultural UniversityHefei, China

**Keywords:** pear, class III peroxidase, gene duplication, microsynteny, expression

## Abstract

Peroxidases (PRXs) are widely existed in various organisms and could be divided into different types according to their structures and functions. Specifically, the Class III Peroxidase, a plant-specific multi-gene family, involves in many physiological processes, such as the metabolism of auxin, the extension and thickening of cell wall, as well as the formation of lignin. By searching the pear genome database, 94 non-redundant *PRXs* from *Pyrus bretschneideri* (*PbPRXs*) were identified. Subsequently, analysis of phylogenetic relationships, gene structures, conserved motifs, and microsynteny was performed. These *PbPRXs* were unevenly distributed among 17 chromosomes of pear. In addition, 26 segmental duplication events but only one tandem duplication were occurred in these *PbPRXs*, implying segmental duplication was the main contributor to the expansion of the *PbPRX* family. By the Ka/Ks analysis, 26 out of 27 duplicated *PbPRXs* has experienced purifying selection. Twenty motifs were identified in PbPRXs based on the MEME analysis, 11 of which were enriched in pear. A total of 41 expressed genes were identified from ESTs of pear fruit. According to qRT-PCR, the expression trends of five *PbPRXs* in subgroup C were consistent with the change of lignin content during pear fruit development. So we inferred that the five *PbPRXs* were candidate genes involved in the lignin synthesis pathway. These results provided useful information for further researches of *PRX* genes in pear.

## Introduction

Pathogenesis related protein (PR) is a type of stress-responsive protein whose expression could be induced by pathogen invasion. According to their amino acid sequences, structures and functions, the PRs could be classified into 17 subfamilies, including Class III Peroxidase (PRX) subfamily (Van Loon et al., 2006; Almagro et al., 2009). As we know, PRXs are widely existed in all kinds of animals, plants and microorganisms. The previous study showed that plants have two kinds of peroxidases, i.e., Class I PRX and Class III PRX (Cosio and Dunand, 2010). Class I PRX contains ascorbate peroxidase (APx) and glutathione peroxidase (GPx), while Class III PRX is a large gene family, including a plurality of members. These Class III Peroxidases members are involved in two possible catalytic cycles, and maintaining both peroxyl radicals and H_2_O_2_ at a stable level in plants. Moreover, they could be extensively involved in many physiological processes of plants, such as resistance to pathogen infection, formation of lignin, auxin decomposition metabolism, seed germination and aging, etc. (Passardi et al., 2004; Almagro et al., 2009; Cosio and Dunand, 2009; Kwon et al., 2015). The numbers of Class III PRX family in *Arabidopsis thaliana, Zea mays*, and *Populus* are 73, 107, and 93, respectively (Almagro et al., 2009; Ren et al., 2014; Wang et al., 2015b). Current research showed that 44 Class III PRX genes in *Arabidopsis thaliana* might be involved in one or more specific mechanisms. For example, *AtPrx21, AtPrx34, AtPrx37, AtPrx52, AtPrx59, AtPrx69*, and *AtPrx71*, could resist to the infection of *Pseudomonas syringae* (Mohr and Cahill, 2007). And 17 Class III PRX genes appeared to decline in *A. thaliana* under microgravity condition, 12 genes of which accumulated in root hairs to participate in the biosynthesis of root hair cell wall, oxidative stress and root development process (Bruex et al., 2012; Petricka et al., 2012; Lan et al., 2013; Kwon et al., 2015). In addition, Ehlting et al. (2005) found that 8 *PRX* genes (*At2g37130, At5g42180, At4g37530, At4g37520, At1g30870, At2g43480, At3g28200*, and *At5g05340*) were involved in the biosynthesis of lignin in *A. thaliana*.

The *PRX* gene family has been well studied in several model plants, such as *Arabidopsis thaliana* (Tognolli et al., 2002; Welinder et al., 2002), *Populus trichocarpa* (Ren et al., 2014), and *Zea mays* (Wang et al., 2015b). However, to date, no previous systematic study of this gene family has been conducted in Chinese pear. In this study, a genome-wide analysis of *PRX* gene family from Chinese pear (*Pyrus bretschneideri*) was conducted via genomic sequence, including *PbPRX* gene models, phylogenetic relationship, genomic structure, chromosome location and other structural features. Furthermore, the expression patterns of 41 *PbPRX* genes during fruit development of pear were also analyzed based on the quantitative real-time PCR (qRT-PCR). The obtained results will establish a solid foundation for further research of the functional roles of *PRX* genes in Chinese pear.

## Materials and methods

### Identification and collection of PRX proteins

In present study, the pear genome database was downloaded from the Pear Genome Project (http://peargenome.njau.edu.cn/), peach was obtained from the Phytozome database (https://phytozome.jgi.doe.gov/pz/portal.html), yang mei was downloaded from the GigaDB database (http://gigadb.org/site/index), and strawberry was obtained from the GDR database (http://www.rosaceae.org/). Subsequently, to identify and annotate PRX proteins in Rosaceae (pear, peach, yang mei, and strawberry), the following approaches were performed. (i) The Hidden Markov Model (HMM) profile of PRX (PF00141) was obtained from the Pfam database (http://pfam.sanger.ac.uk/search) (Punta et al., 2011). (ii) The HMM profile was used to survey all pear proteins by DNAtools software. (iii) To confirm the reliability of searched results, all candidate protein sequences were checked in SMART (Letunic et al., 2012) and Pfam (Punta et al., 2011). (iiii) By sequence alignment and chromosomal location analysis, all non-redundant PRX proteins were identified for further analysis.

### Phylogenetic analysis

For the phylogenetic tree of PbPRX, 94 full-length PRX proteins were aligned using ClustalX2.1 (http://clustalx.software.informer.com/2.1/). By the alignments, an unrooted neighbor-joining (N-J) tree was constructed by MEGA 7.0 software (Kumar et al., 2016) with bootstrap analysis (1000 replicates). For the composite phylogenetic tree, PRX proteins from peach, yang mei, strawberry, and Chinese pear were similarly aligned with ClustalX 2.1. Phylogenetic trees were generated using the neighbor-joining (N-J) method with MEGA 7.0 software (Kumar et al., 2016).

### Gene structural and conserved motif analysis

Genomic sequences of Chinese pear v.1.0 annotation were obtained from GigaDB database (http://gigadb.org/site/index). CDS sequences were aligned to DNA sequences and schematics generated using Gene Structure Display Server (http://gsds.cbi.pku.edu.cn) (Guo et al., 2007). Sequences of the 94 PbPRX proteins were analyzed using online MEME website (http://meme-suite.org/tools/meme) (Bailey et al., 2015) with the following parameters: minimum motif width, 6; maximum motif width, 200; maximum number of motifs, 20. The conserved motifs were annotated using the SMART database (Letunic et al., 2012) and Pfam database(Punta et al., 2011). HMMs were constructed from the MEME alignment of each motif using DNAtools software. PRX protein sequences of *Arabidopsis thaliana, Zea mays, Populus*, and *Vitis vinifera* retrieved from the TAIR database (http://www.arabidopsis.org/) and Phytozome database (https://phytozome.jgi.doe.gov/pz/portal.html) were searched with the HMMs using DNAtools software.

### Chromosomal localization and test for selection neutrality

Chromosomal linkage visualization was proformed by MapInspect software (http://mapinspect.software.informer.com/). Synonymous (Ks) and nonsynonymous (Ka) substitution rates were calculated by DnasP v5.0 (Librado and Rozas, 2009). The selection modes of *PbPRX* paralogs were determined by analyzing the Ka/Ks ratios. Subsequently, a sliding window analysis of nonsynonymous substitutions per nonsynonymous site Ka/Ks ratios was conducted with the following parameters: window size, 150 bp; step size, 9 bp.

### Interspecies microsynteny analysis

To detect the syntenic regions among peach, yang mei, strawberry and Chinese pear, the MCScanX software (Wang et al., 2012) was used. Subsequently, the duplicated *PRXs* were identified in these syntenic regions, as well as the relationships of *PRX* orthologous pairs among peach, yang mei, strawberry and Chinese pear were plotted using Circos software (Krzywinski et al., 2009).

### Expression analysis by ESTs

Chinese white pear EST sequences were downloaded from Pear Genome Project (http://peargenome.njau.edu.cn/). *PbPRX* genes were used as query to blast against Chinese pear ESTs using BLASTN and the parameters were set as follows: *E*-value < 10e−10, length > or = 200 bp, and maximum identity >95%.

### Plant material

The Chinese pears, picked from 40-year-old pear trees grown on a pear orchard (Dangshan, Anhui, China). Ten robust and healthy trees that were managed in a consistent manner were selected. In April 2015 (i.e., when the trees were at the bud stage), short branches bearing buds of similar developmental stages and sizes were selected from the mid-crown area on the south side of each tree, and then labeled. Only two fruits were kept for each short branch. Fruits were picked every 8 days from 15 days after flowering. Forty uniformly sized fruits were collected at each time, refrigerated, and transferred to the laboratory for further experiment.

### RNA extraction and qRT-PCR

In present study, eight fruit samples at 15days after flowering (DAF), 39 DAF, 47 DAF, 55 DAF, 63 DAF, 79 DAF, 102 DAF, and 145 DAF were collected for quantitative real-time PCR (qRT-PCR) analysis. We used the RNAprep pure Plant Kit (Tiangen, Beijing) to extract total RNA from all the samples based on the manufacturer's instructions. Subsequently, about 1 μg of total RNA was used for reverse transcription using PrimeScript™ RT reagent Kit with gDNA Eraser (Takara, Japan). We designed gene-specific primers (Table S1) to amplify 41 *PbPRX* genes using Beacon Designer 7 software. qRT-PCR analysis was carried out Bio-rad CFX96 Touch™ Deep Well Real-Time PCR Detection System using SYBR® Premix Ex Taq™ II (Takara, Japan) based on the manufacturer's protocol. Pyrus tubulin gene (Wu et al., 2012) was used as internal control gene and the relative expression levels of these *PbPRXs* were estimated by the 2^−ΔΔCT^ method (Livak and Schmittgen, 2001). The statistical analyses were performed using SPSS20.0 software. The data were presented as mean value ± standard error. Student's *t*-test was used to illuminate the statistical analysis of significant differences between controls (15 DAF) and the treatments.

### Determination of the lignin content of pear

According to Syros et al. (2004), 0.02 g dry powder of pear stone cell was transferred into the 10 mL grinding mouth tube, and then 2 mL 25% acetyl bromide-glacial acetic acid (w/w) and 0.1 mL of perchloric acid were add, followed by the water bath for 30 min at 70°C (oscillation every 10 min). After the dry powder dissolved, 3 mL 2 mol/L NaOH was added and the mixture was transferred to the centrifuge tube, and the supernatant was fixed volume to 50 mL with ice acetic. The absorbance value (ABS) was measured at 280 nm, 3 repeats.

## Results

### Identification of *PRX* genes in chinese pear

To identify members of PRX family, HMM searches were performed against the pear genome database with the BLASTP program. A total of 101 candidate PRX proteins were identified in Chinese pear. To identify the members of PRX proteins family, the proteins were checked for the presence of PRX domain by Pfam database (Punta et al., 2011) and SMART database (Letunic et al., 2012). Four of 101 candidate PRX proteins were discarded in this study because of the lacking of PRX domain or complete PRX domain. In addition, 3 redundant PRX proteins were excluded based on the sequences similarity analysis. All other 94 non-redundant *PbPRXs* were named as *PbPRX1*-*PbPRX94*, and used for further analysis. Their detailed information, including chromosome location, gene name and gene identifier, was listed in Table S2.

### Phylogenetic analysis of the *PRX* genes in four species from rosaceae

**Figure 1:**
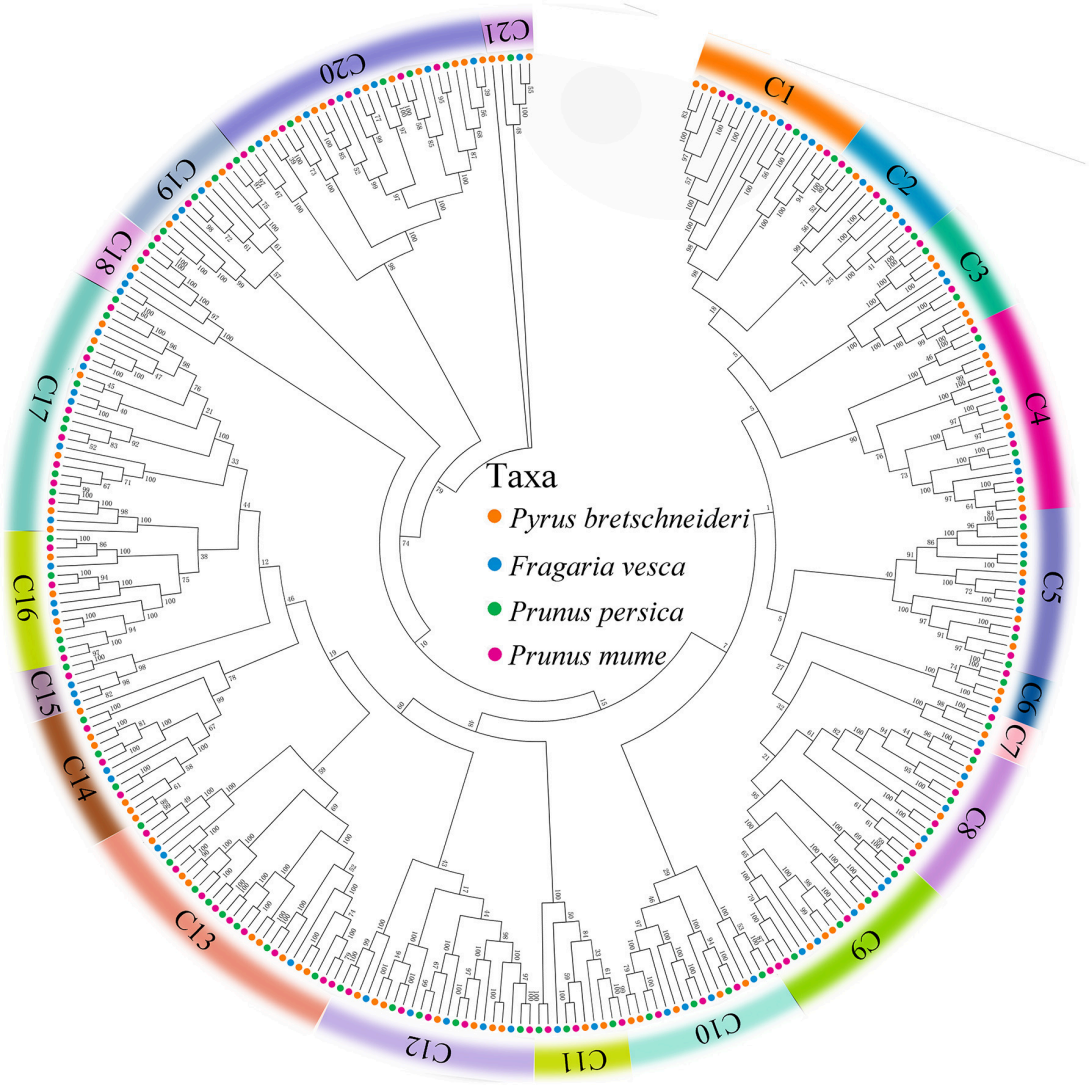
**Phylogenetic relationships and subfamily designations in PRX proteins from *Pyrus bretschneideri* Rehd, *Prunus mume, Prunus persica*, and *Fragaria vesca***. Theneighbor-joining tree includes 94 PRX proteins from *Pyrus bretschneideri* Rehd, 76 from *Prunus mume*, 74 from *Prunus persica* and 73 from *Fragaria vesca*. These proteins were divided into 21 Clades (C1–C21), and were represented by different colors, respectively.

To investigate the evolutionary relationships of the *PRX* gene family in peach, yang mei, strawberry, and Chinese pear, we used all of the *PRX* genes from peach (74), yang mei (76), strawberry (73), and Chinese pear (94) to construct an Neighbour-Joining (N-J) phylogenetic tree by MEGA 7.0 software (Kumar et al., 2016). Bootstrapping tests were performed on this tree. Based on the bootstrap values and the topology of the tree, all of the PRX proteins were divided into 21 clades (Figure 1 and Figure S1), designated as clade 1 to clade 21, with strong bootstrap values. The results showed that there was not equal representation of peach, yang mei, strawberry, and Chinese pear *PRX* genes within given clades (C1-C21). For instance, the C7 subfamily includes four peach *PRX* genes, five yang mei *PRX* genes, five strawberry *PRX* genes, and includes six Chinese pear *PRX* genes (Figure 1). In contrast, phylogeny subfamily C15 includes three strawberry *PRX* genes, one peach *PRX* gene, and one yang mei *PRX* genes, but not one pear *PRX* gene. Remarkably, we found that *PRX* genes from peach and yang mei show close pairwise relationships based on genetic distance, compared with other proteins from different species (Figure 1 and Figure S1).

Interestingly, we found that each of the four Rosaceae species (peach, yang mei, strawberry, and Chinese pear) contributed at least one *PRX* gene to each clade by a strong bootstrap support, except clade 15. This result implied that rapid duplication of *PRX* genes occurred before these dicotyledon species diverged (Figure 1 and Figure S1).

### Phylogenetic, structural, and conserved motifs analyses of PRX proteins in pear

**Figure 2:**
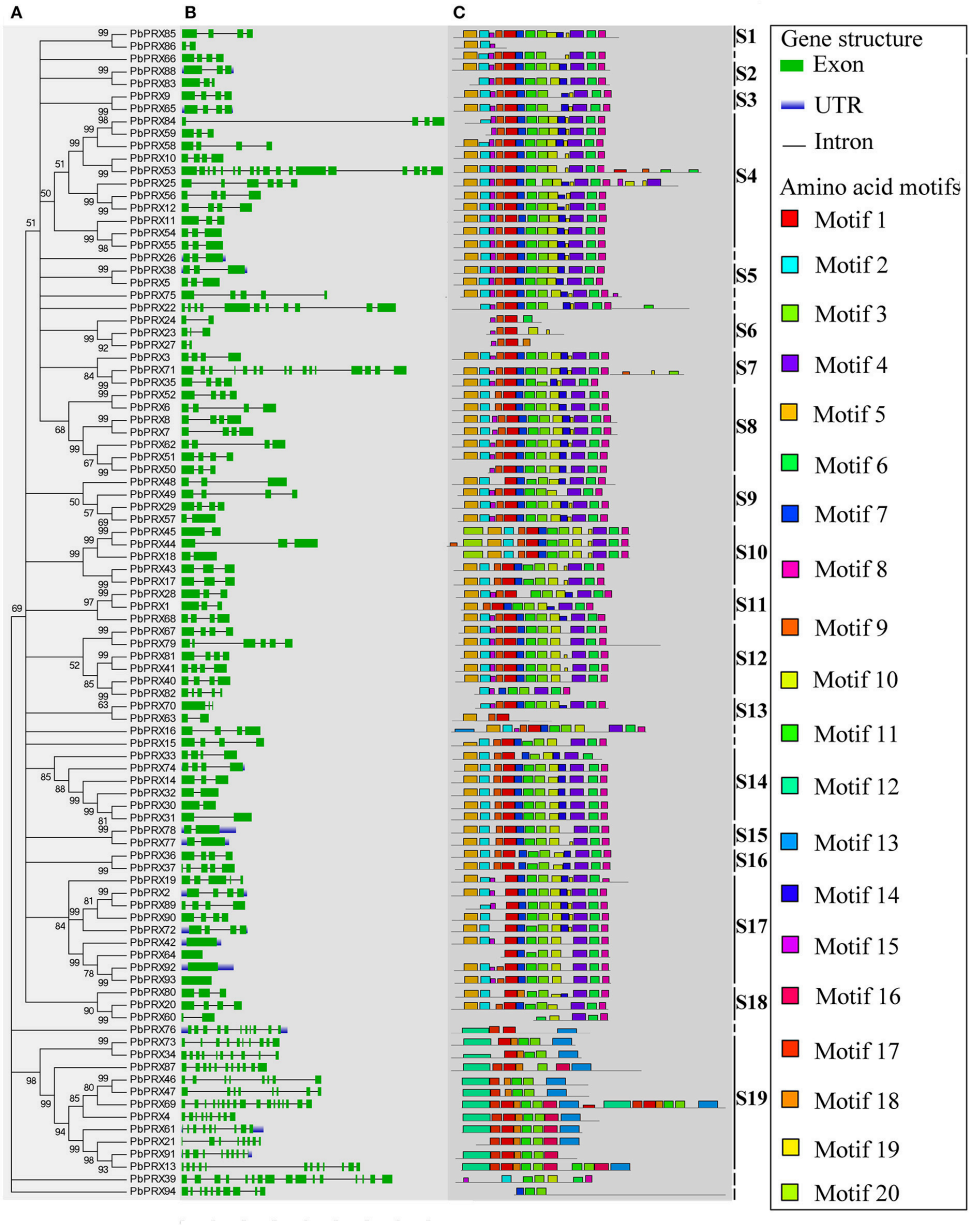
**Predicted *Pyrus bretschneideri* Rehd PRX (PbPRX) protein phylogeny, conserved motifs and exon–intron structure. (A)** Unrooted Neighbour-Joining phylogeny of PbPRXs, with bootstrap values>50. **(B)** Exon/intron organization of *PbPRX* gene models. **(C)** Distribution of amino acid motifs in pear PRX proteins. Exons, introns, and untranslated regions (UTRs) are indicated by green boxes, gray lines and blue lines, respectively. Relative protein or gene lengths can be estimated by gray bars.

A phylogenetic tree of 94 PbPRX proteins in pear was constructed using Neighbour-Joining method. By phylogenetic analysis, these PbPRXs were further divided into 19 subfamilies (Figure 2A), designated as S1 to S19, with strong bootstrap values. The subfamily S4 and S19 are of the largest two groups with 11 members. On the contrary, subfamily S1, S2, S3, S5, S13, S15, and S16 only had two members (Figure 2A).

**Figure 3:**
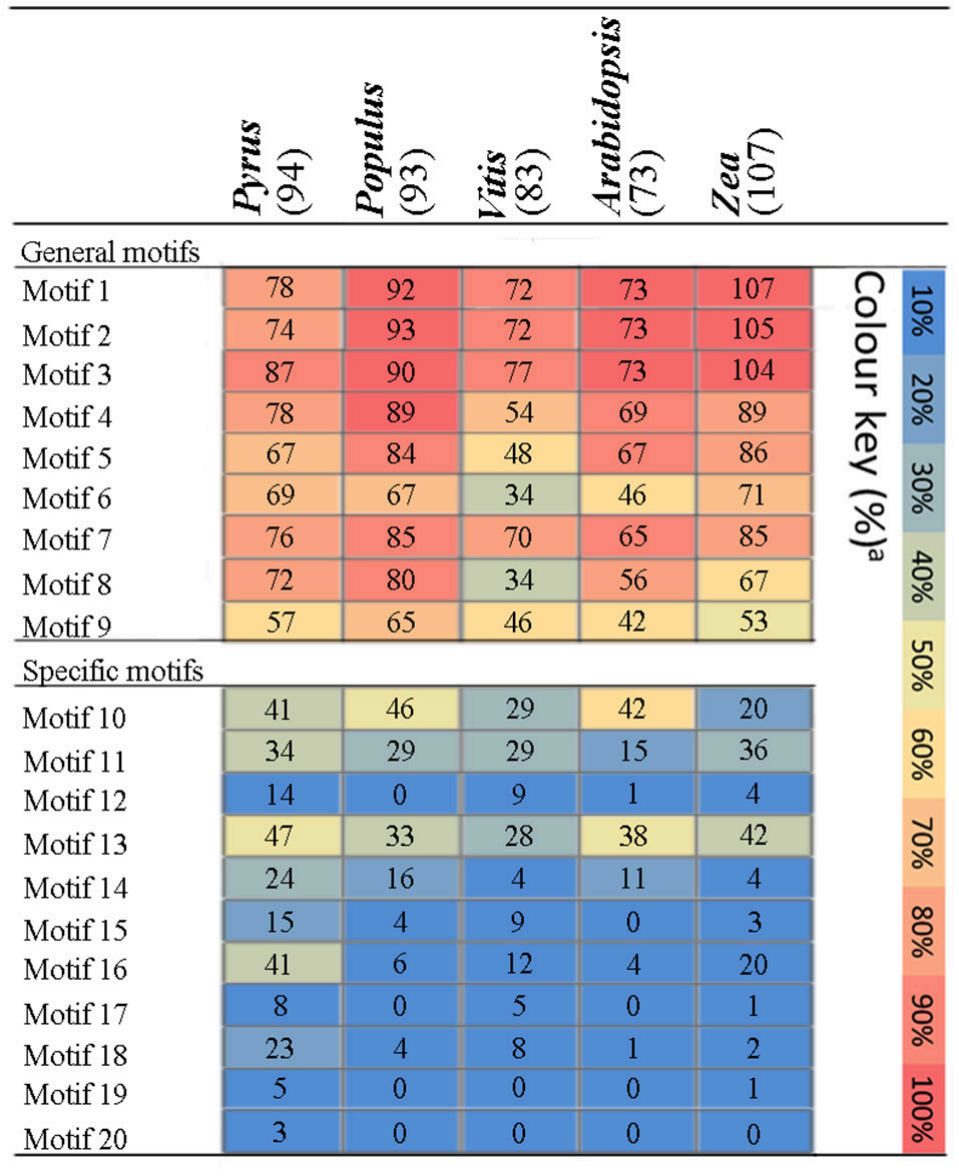
**Distribution of amino acid motifs in PRX proteins from *Pyrus bretschneideri* Rehd, *Populus trichocarpa*, *A*rab*idopsis thaliana*, *Vitis vinifera*, and *Zea mays***. Colure key: The percentage of PRX sequences in each genome containing a particular motif was represented by a heat map.

To understand their functional regions, conserved motifs analyses of PbPRX proteins were performed. Twenty conserved motifs (Table S3) with 6-200 residues in the 94 PbPRX proteins were identified using the MEME tool (Bailey et al., 2015). Motif composition and arrangement were in good agreement with the phylogenetic tree (Figure 2C). The arrangement and composition of the motifs were basically consistent with the results of the phylogenetic tree (Figure 2). Using Hidden Markov Models (HMMs) describing subdomains A-F of the PRX protein based on the previous approaches (Ooka et al., 2003; Hussey et al., 2015), we assigned Motif 1, Motif 2, Motif 3, and Motif 4 to subdomain A, Motif 5, Motif 6, Motif 7, Motif 8, and Motif 9 to subdomain B, subdomain C, subdomain D, subdomain E, and subdomain F, respectively. In addition, we found that these motifs occurred in the N-terminal half of PbPRX proteins (Figures 2, 3). Due to their distribution in most (>65%) of the PbPRX proteins, these motifs were called “general motifs.” The remaining Motif 10–20 were classified as “specific motifs” that were restricted to 3–47 PbPRX proteins (Figure 3). Specific motifs were occurred in the diverse C-terminal region with unknown function (outside the PRX domain), based on both annotation of SMART (Letunic et al., 2012), and Pfam databases (Punta et al., 2011).

The distribution of these motifs in other plant genomes was further evaluated by HMMs that were obtained from the pear alignments of each motif (Table S3). The matching motifs were identified from the PRX pronteins of *Populus* (Ren et al., 2014), *A. thaliana* (Livak and Schmittgen, 2001), *Vitis vinifera*, and *Zea mays* (Wang et al., 2015b), using pear as a positive control. The general motifs were enriched in PRX proteins from the genomes tested (Figure 3). By contrast, compared with other genomes, the specific motifs of the PRX protein were detected at high frequencies in pear, with the exception of Motif 10 and Motif 11. Motif 17 only exist in pear, *Vitis vinifera* and *Zea mays* (Figure 3), implying that it might be related to the maturation or quality of fruits. Interestingly, Motif 20 was exclusively found in PbPRX proteins and may thus represent motifs unique to pear (Figure 3). Compared to other genomes, there was no bias observed in the cumulative frequency of general motifs identified in pear, so pear-specific motifs were an artifact of HMMs built on pear alignments is impossible.

Then, exon-intron analysis was performed in *PbPRX* genes (Figure 2B). The results revealed that, there were no introns in *PbPRX42, PbPRX64, PbPRX92*, and *PbPRX93*, whereas in the others the intron number was ranged from 1 to 17 (Figure 2B). Additionally, both numbers of intron and exon were well conserved between closely related genes. These results were consistent with the previously reported for maize *PRX* genes (Wang et al., 2015b).

### Interspecies microsynteny analysis

**Figure 4:**
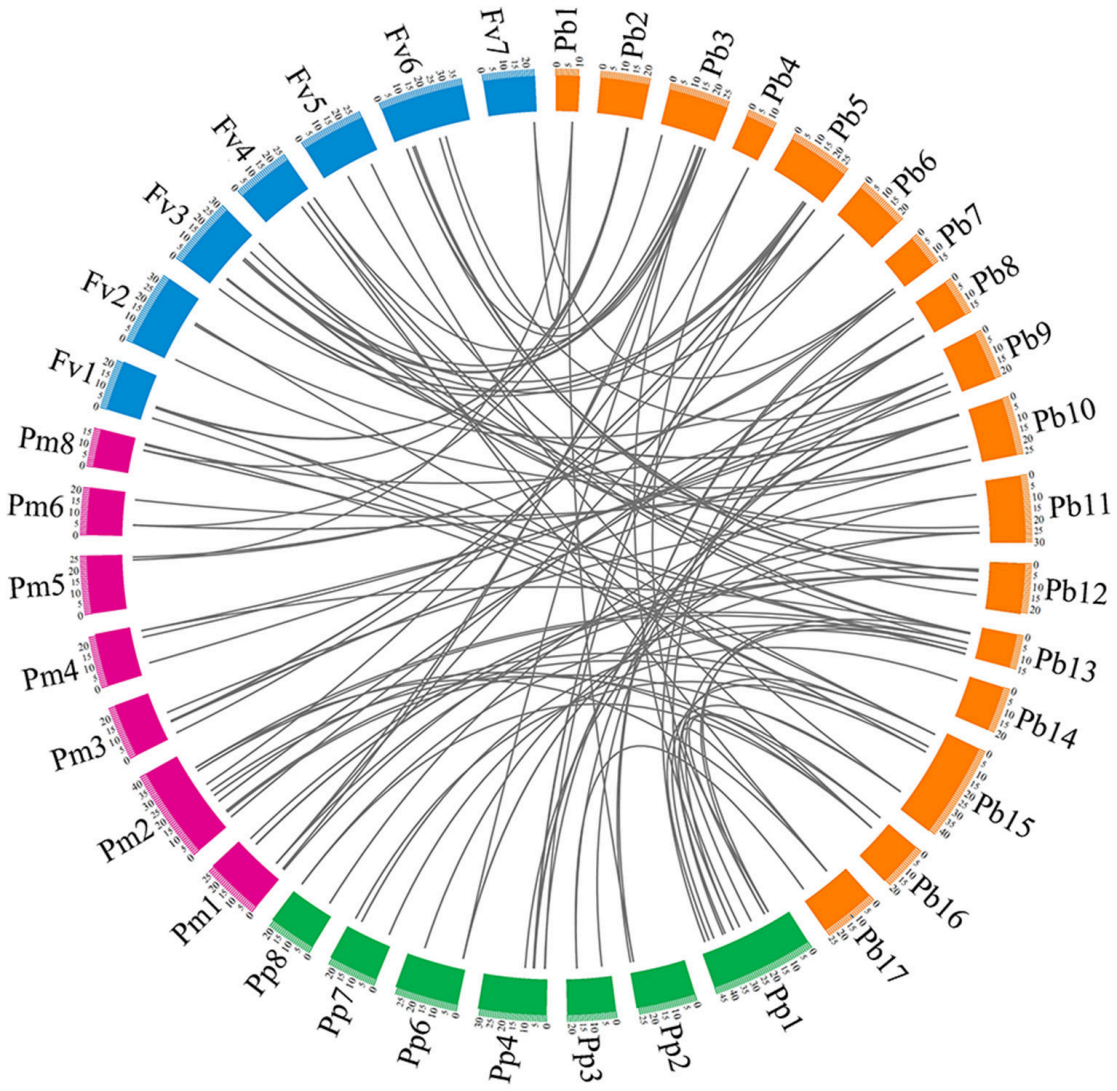
**Microsynteny of PRX regions across pear, yang mei, peach, and strawberry**. The pear, yang mei, peach and strawberry chromosomes are shown in different color boxes, as well as labeled Pb, Pm, Pp, and Fv, respectively. Syntenic relationships between PRX regions are represented by gray lines.

To understand the evolutionary relationship of the *PRX* gene family among peach, yang mei, strawberry, and Chinese pear, interspecies microsynteny analysis was conducted to identify orthologous *PRX* genes (Figure 4 and Table S4). Our results show that pear and yang mei shared the most orthologous pairs, of up to 46 pairs of orthologous *PRXs*, followed by pear and peach (41). However, we only identified 39 pair of orthologous *PRXs* between pear and strawberry (Figure 4 and Table S4). These results may reflect the closer relationship between pear and peach/yang mei vs. pear and strawberry. Additionally, we found some orthologous gene pairs between pear and peach/yang mei, and were not found between pear and strawberry (Figure 4 and Table S4), such as *PbPRX29*/*ppa023401m*/*Pm019665, PbPRX33*/*ppa008667m*/*Pm004069, PbPRX36*/*ppa025451m*/*Pm005657*, indicating these orthologous pairs appeared after strawberry diverged from the common ancestor of pear and peach/yang mei. Interestingly, we found a series of two or more matched one *PRX* gene between pear and peach/yang mei/strawberry. We speculated that these genes were paralogous gene pairs and played a key role in the expansion of the *PRX* gene family in the process of evolution. For instance, *Pm026922* and *Pm020853* are orthologous genes to *PbPRX4, ppa023088m*, and *ppa008516m* are orthologous genes to *PbPRX41*, as well as *mrna18099* and *mrna25391* are orthologous genes to *PbPRX13* (Figure 4 and Table S4).

### Physical distribution of pear *PRX* genes

**Figure 5:**
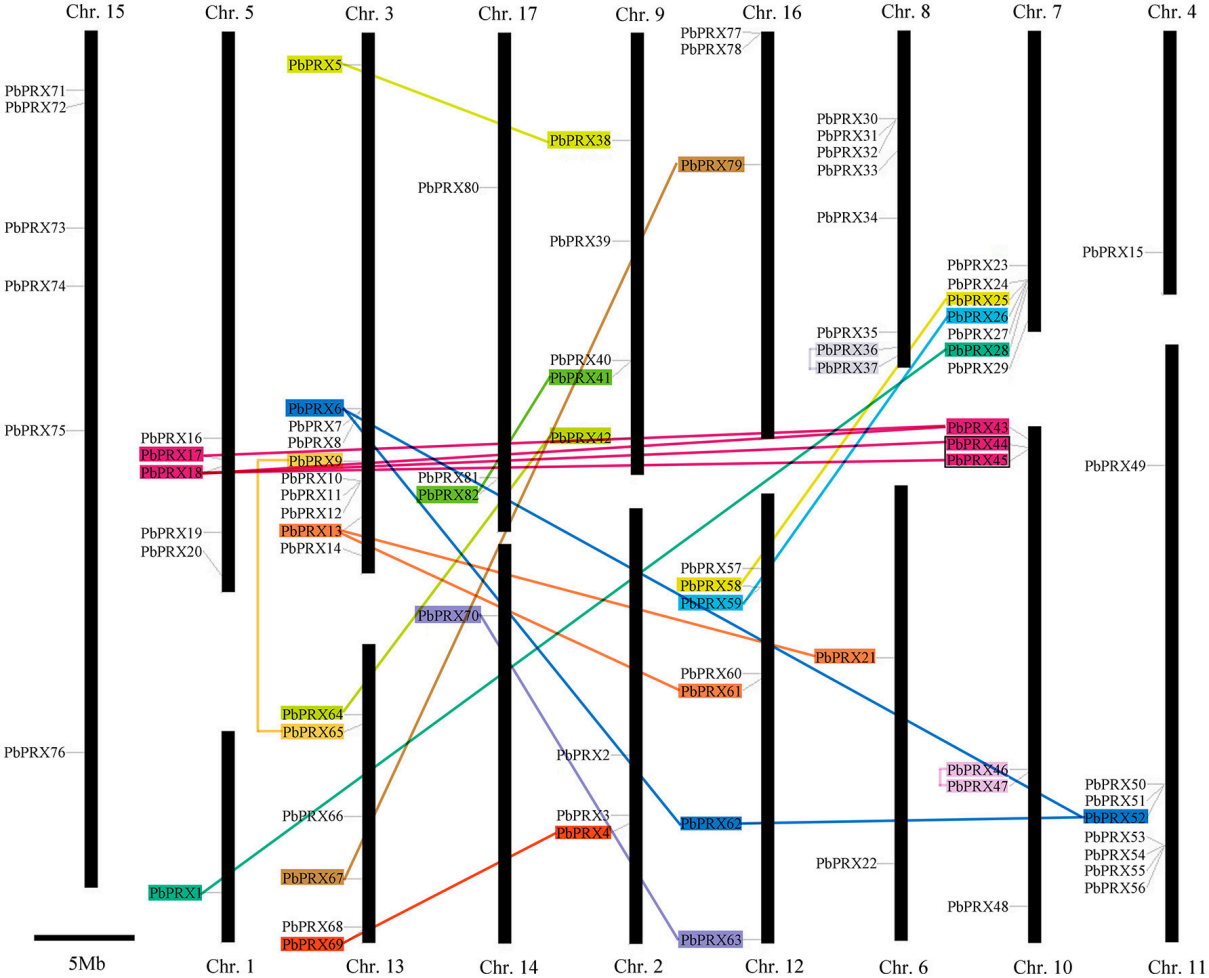
**Chromosomal locations of *Pyrus bretschneideri* Rehd PRX (*PbPRX*) genes**. Segmental duplicates represented by color boxes and connected by color lines, and tandem duplicates are marked by black sides.

In our research, 94 *PbPRXs* were distributed among 17 chromosomes or scaffolds, based on their positions in the pear genome. Chromosome 3 contained highest numbers of *PbPRXs* (10), followed by Chromosome 8 (8) and Chromosome 11 (8). By contrast, there presented only one *PbPRX* on both chromosome 1 and chromosome 4 (Figure 5). In addition, higher density of *PbPRXs* was found on some regions of chromosomes, such as the top of chromosome 10, and the bottoms of chromosomes 3, 7, 11, respectively. The *PbPRX* family has undergone through many processes, including tandem duplication, segmental duplication, or whole-genome duplication. To further understand how *PbPRX* genes were evolved, gene duplication events were investigated in pear. In present study, 26 gene pairs were arranged in blocks of segmental duplicate and only one gene pairs (*PbPRX44*/*PbPRX45*) were arranged in blocks of tandem duplicate (Figure 5). These results strongly implied that segmental duplication events were the major contributors to the expansion of the pear *PRX* family, but tandem duplication events. However, in previous studies, segmental duplication (16) and tandem duplication (12) were identified in maize *PRX* family (Wang et al., 2015b). There was a significant difference with the expansion pattern of the *PRX* genes in maize and pear, which strongly implied that *PRX* family members of different species have different expansion pattern. It might be the reason why the *PRX* family members (94) in pear were less than those in the maize (119) (Wang et al., 2015b).

### Strong purifying selection for *PRX* genes in pear

**Figure 6:**
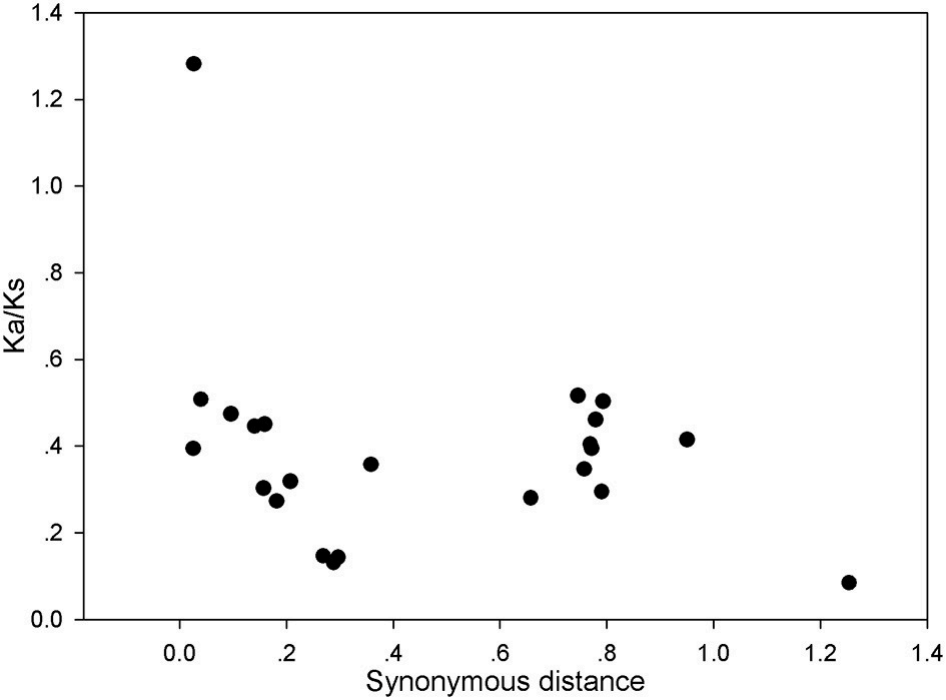
**Scatter plots of the Ka/Ks ratios of duplicated PRX genes in pear**. X-axis represents the synonymous distance, and y-axis the Ka/Ks ratio for each pair, respectively.

The above results indicate that the gene duplication events were the main contributor to the expansion of the *PRX* gene family in pear. To explore the selection pressures acting on this gene family, Ka, Ks, and Ka/Ks ratios were calculated for the 27 gene pairs. Generally, Ka/Ks < 1 indicates purifying or negative selection, Ka/Ks = 1 stood for neutral selection and Ka/Ks > 1 stood for positive selection. In this study, all the Ka/Ks values from the 26 pairs of pear *PRX* gene were less than 0.6, with the exception of*PbPRX36*/*PbPRX37* (Figure 6 and Table S5). So we proposed that the *PRX* gene family had mainly undergone strong purifying selection, with the slowly evolving at the protein level of this gene family. Subsequently, the dates of the duplication events were estimated according to estimations for Ks. As a result, the duplicated events in pear were calculated to have occurred at about 8 to 84.3 million years ago (Table S5).

In the process of positive selection, some codon sites might be masked by the overall strong negative selection. Therefore, a sliding-window analysis of Ka/Ks ratios were performed between each pair of the duplicated *PbPRX*. As expected, numerous sites/regions were under neutral to strong purifying selection based on the sliding window analysis of Ka/Ks values (Figure S2). Meanwhile we also clearly found that most of Ka/Ks ratios across coding regions were far less than 1, except for one or several distinct peaks (Ka/Ks > 1). Interestingly, the conserved domains of *PbPRX* have undergone strong purifying selections (Ka/Ks < 1). Eight exception (*PbPRX6*/*PbPRX52, PbPRX9*/*PbPRX65, PbPRX17*/*PbPRX43, PbPRX42*/*PbPRX84, PbPRX36*/*PbPRX37, PbPRX44*/*PbPRX45, PbPRX59*/*PbPRX84*, and *PbPRX67*/*PbPRX79*) with Ka/Ks ratios >1 in sites of their *PRX* domains (Figure S2), suggested a positive selection in this region, indicating these genes undergone slightly different selection pressure. Moreover, positive selection could result in a higher ratio of Ka/Ks. However, it could not guarantee that the average Ka/Ks ratio of the gene would be more than one. These results strongly suggested that purifying selection might play a key role in the evolution of the *PRX* gene family in pear.

### Expression characteristics of pear *PRX* genes

To further explore *PRX* gene functions in pear, expression of *PbPRX* genes during fruit development were investigated. First, the *PbPRX* genes were searched for in the pear expressed sequence tags (ESTs) database. 41 out of 94 *PbPRX* genes were found to have EST hits (Table S6). A total of 96 EST hits were found for all *PbPRX* genes, and *PbPRX20* contained highest number of EST hits (44), followed by *PbPRX59* (4). However, 53 *PbPRX* genes were not searched any EST hits in the EST database, revealing the functions of these genes need to be further studied at other time points or other tissues.

**Figure 7:**
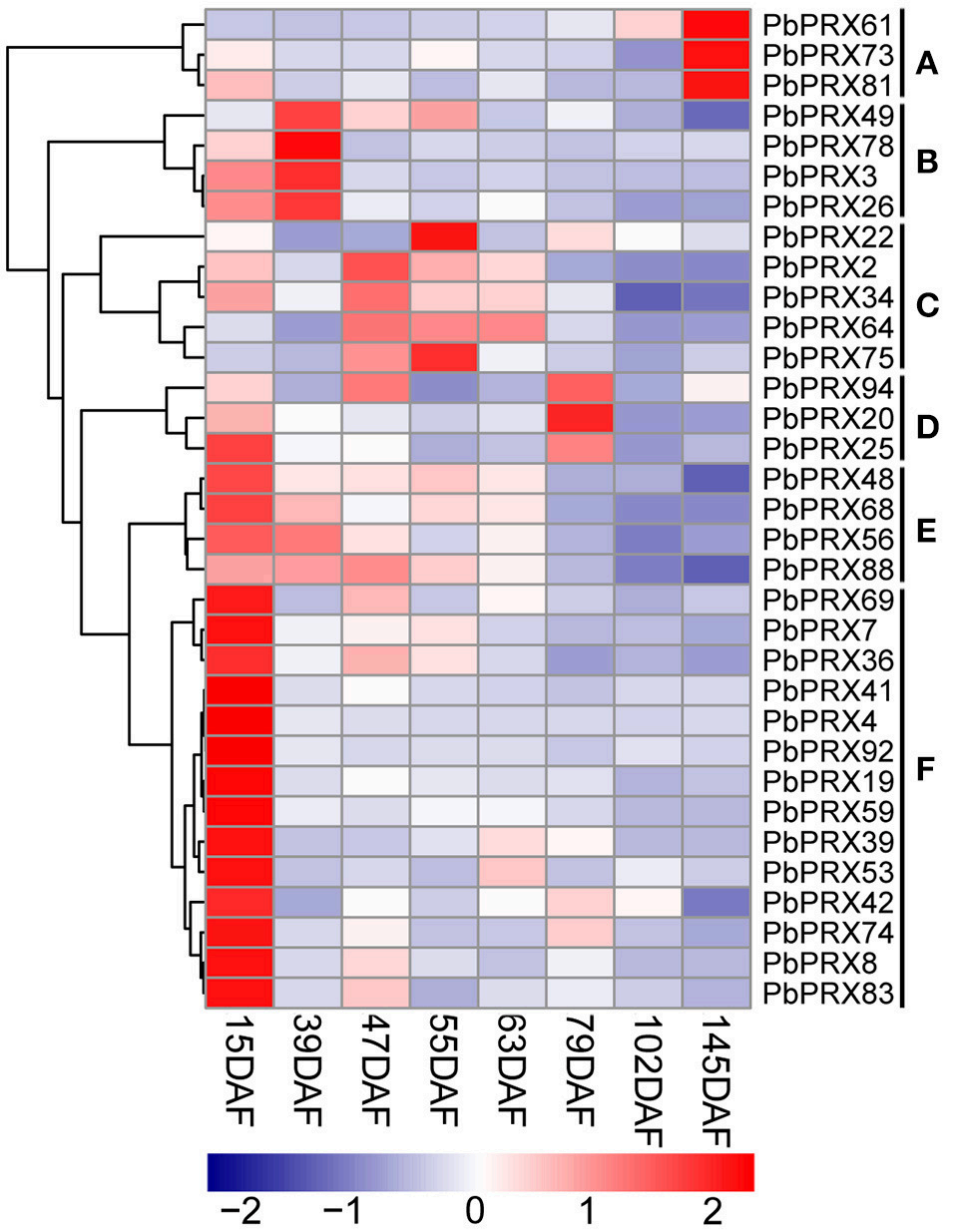
**Heat map representation of *PbPRX* genes the eight stages of pear fruit development, 15 days after flowering (DAF), 39 DAF, 47 DAF, 55 DAF, 63 DAF, 79 DAF, 102 DAF, and 145 DAF**. These expression profile data were obtained using qRT-PCR, and relative expression was log2 transformed, thereby values −2, −1, 0, 1, and 2 represent low, intermediate and high expression, respectively.

Then, qRT–PCR was conducted using fruit samples (15 DAF, 39 DAF, 47 DAF, 55 DAF, 63 DAF, 79 DAF, 102 DAF, and 145 DAF). 33 out of 41 *PbPRX* genes showed a variety of expression patterns during different developmental stages of pear fruit (Figure 7). According to the expression profiles, the *PbPRXs* can be divided into six subgroups (A–F). This result revealed that most subgroups have similar expression patterns (Figure 7). Three genes from subgroup A (*PbPRX61, PbPRX73*, and *PbPRX81*) showed the highest transcript level at 145 DAF; four from subgroup B (*PbPRX3, PbPRX26, PbPRX49*, and *PbPRX78*) at 39 DAF; three from subgroup D (*PbPRX20, PbPRX25*, and *PbPRX94*) at 79 DAF; eighteen from subgroup E (*PbPRX*48, *PbPRX56, PbPRX68*, and *PbPRX88* and subgroup F: *PbPRX4, PbPRX7, PbPRX8, PbPRX19, PbPRX36, PbPRX41, PbPRX42, PbPRX53, PbPRX59, PbPRX69, PbPRX74, PbPRX83*, and *PbPRX92*) at 15 DAF, indicating that these genes might play important roles in the early stages, or middle stages, or mature stage of pear fruit development.

**Figure 8:**
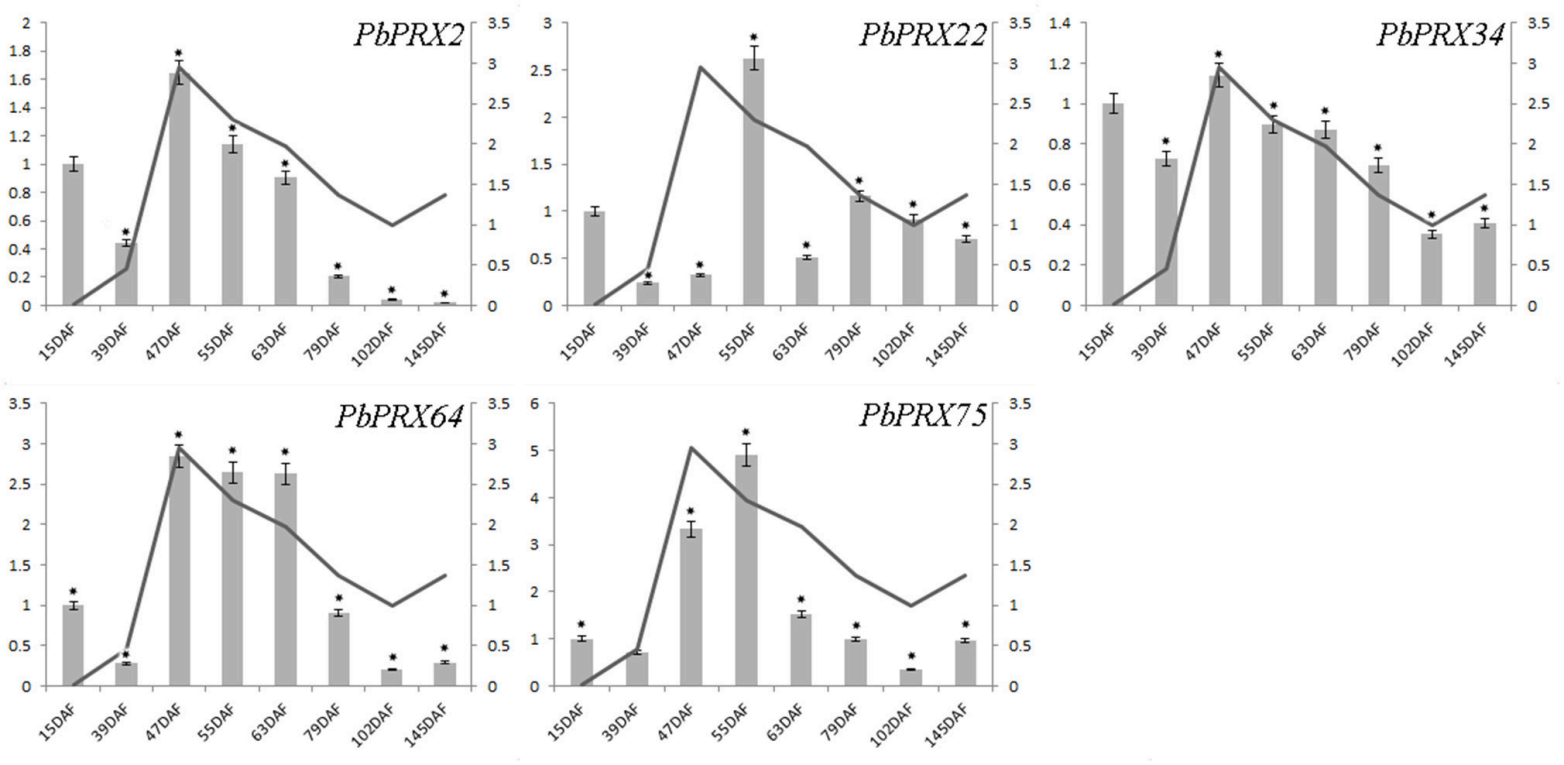
**Comparison of relative expression levels of five *PbPRXs* involved in lignin synthesis and content of stone cells during fruit development in *Pyrus bretschneideri***. Y- axes on the left indicates the relative gene expression levels:15, 39, 47, 55, 63, 79, 102, 145 DAF (X-axis) by bar charts, and the Y- axes on right showed the content of stone cells during fruit development with line charts. Each histogram represents the mean value and the bar ± standard error of three biological replicates. ^*^significant difference at *P* < 0.05.

The content of stone cells was an important factor to affect the quality of pear fruit. As one of main components in stone cell walls, lignin synthesis had a direct impact on the formation of stone cells which are enriched in pear fruit (Cai et al., 2010; Jin et al., 2013). In addition, the change of lignin content was also correlated with that of stone cell content (Cai et al., 2010; Jin et al., 2013). In this study, we found the lignin content was relatively low at the beginning of pear fruit development, then reached a peak at the middle stage, and gradually decreased at mature stage (Figure 8). Interestingly, five genes from subgroup C (*PbPRX2, PbPRX22, PbPRX34, PbPRX64*, and *PbPRX75*) showed the tendency with the highest transcription accumulation at 47, 55, or 63 DAF (that is at the middle stage), which were consistent with physiological indicators of lignin in pear fruit. Therefore, it was postulated that these genes should be considered as putative candidate genes involved in regulation of the lignin synthesis pathway for pear fruit.

## Discussion

Members of Class III Peroxidases gene family are involved in the regulation of a variety of processes (Tognolli et al., 2002; Ren et al., 2014; Wang et al., 2015b). Systematic and comprehensive analyses of *PRX* gene families have been published for *Populus trichocarpa* (Ren et al., 2014), *Zea mays* (Wang et al., 2015b), *Arabidopsis thaliana* (Tognolli et al., 2002), and *Oryza sativa* (Passardi et al., 2004). As the Chinese Pear Genome Project (http://peargenome.njau.edu.cn/) was completed in 2012 (Wu et al., 2013), the genome data of pear (*Pyrus bretschneideri* Rehd.) provides a useful tool for analysis of the pear PRX gene family. In the present study, 94 non-redundant PRX proteins were identified in pear, which consist of one of the largest PRX families known in woody plants. Subsequently, gene structure, chromosomal locations, phylogenic relationships, gene duplication events, and interspecies microsynteny analysis were performed.

To explore the evolutionary relationships of *PRX* gene family in Rosaceae, phylogenetic tree was constructed with 94 non-redundant *PbPRXs*, plus74 *PpPRXs* in peach, 76 *PmPRXs* in yang mei, and 73 *FvPRXs* in strawberry by MEGA software (Kumar et al., 2016) using Neighbour-Joining method, which divided into 21 clades. These results revealed that most of clades contained different numbers of *PRX* genes from pear, peach, yang mei, and strawberry, displaying a relatively conserved evolution in the Rosaceae species tested. In addition, certain divergences have also been found among these species. For example, the *PRXs* genes from pear and yang mei/peach appear to be more closely related to each other than to those from strawberry. This conclusion was supported by the analysis of interspecific microsynteny in pear, peach, yang mei, and strawberry (Figure 4), such as the number of orthologous genes between pear and yang mei (46), pear and peach (41) was higher than that between pear and strawberry (39).

In the process of genome evolution, tandem duplication and segmental duplication were the main factors that led to the expansion of gene family (Cao et al., 2016b). Like *A. thaliana*, maize, and *Populus*, the genome of the Chinese pear has undergone several genome-wide duplication events in early evolution (Wu et al., 2013). A total of 26 *PbPRX* gene pairs were identified as segmental duplication in this study, which accounted for 35% of the total genes. However, only *PbPRX46* and *PbPRX47* were involved in tandem duplication, indicating segmental duplication was the main way to expand *PRX* genes in Chinese pear. In *Populus*, about 38% of the *PRX* genes were involved in segmental duplication, and there were no obvious gene clusters and tandem duplications in the *Populus* genome (Ren et al., 2014). On the other hand, about 26% of the *PRX* genes were involved in the segmental duplication, and 20% of the *PRX* genes were involved in tandem duplication in maize (Wang et al., 2015b). These results showed that segmental duplication might be more significant in both *PRX* genes of poplar (38%) and Chinese pear (35%) than those of maize (26%). These results suggested that the segmental duplication is likely the main reason for the expansion of *PRX* gene family in Chinese pear and poplar. However, in maize, the segmental duplication and tandem duplication almost identically contributed to the *PRX* gene family expansion. These also explain why the number of *PRX* genes in Chinese pear (94) and *Populus* (93) were less than those in maize (119).

Notably, the conserved motif 19 and motif 20 was identified as the pear-specific protein motifs in the C-termini of PbPRXs. They were restricted to specific clades of PbPRX proteins, and although the function remains to be further studied, might play a key role during fruit development of pear. Additionally, gene structures were also investigated in *PbPRX* genes. We found that 94 *PbPRX* genes contained different numbers of exons or introns, implying a great diversity in *PRX* gene family of pear. Many studies have shown that structural diversification of genes plays an important role in the evolution of multi-gene families (Muthamilarasan et al., 2014; Cao et al., 2016a,b,c; Han et al., 2016). In our research, we found that there presented different characteristics of PRXs from different subfamilies, indicating PbPRXs have functionally diversified. In addition, previous studies have reported that introns could be specifically inserted into the plants, as well as were retained in the plant genome during evolution (Rogozin et al., 2003; Wang et al., 2015a). So we inferred that loss or gain of introns may be caused by specific approach. In the present study, we also found this phenomenon, which might explain the functional differences and diversity of closely related *PbPRX* genes, such as *PbPRX7* and *PbPRX8, PbPRX72*, and *PbPRX90* (Figure 2).

In this study, expression patterns of 41 *PbPRXs* during fruit development of pear were tested using qRT-PCR. Among them, the expression levels of five genes (subgroup C: *PbPRX2, PbPRX22, PbPRX34, PbPRX64*, and *PbPRX75*) were lower at early stages, and gradually increased from the early to the middle stage, then decreased during pear fruit ripening, which were significantly correlated to lignin content during fruit development. In addition, previous research have demonstrated that PRX could directly affect the synthesis of lignin via regulation of the last reaction in lignin metabolism pathway (Davin et al., 2008). Therefore, it was suggested that these five genes were participated in regulation of the lignin synthesis in pear fruit. Remarkably, two pairs of segmental duplication genes (*PbPRX4* and *PbPRX69, PbPRX26*, and *PbPRX59*), showed similarly expression levels at eight time points after flowering. Although their expression levels peaked at different time points, both of *PbPRX26* and *PbPRX59* showed the similar tendency throughout pear fruit development.

## Conclusion

In short, a total of 94 full-length *PRX* genes were identified in pear and divided into 19 subfamilies, as supported by intron-exon structures, conserved motifs and phylogeny. Chromosomal mapping and microsynteny analysis suggested that these *PbPRXs* were unevenly distributed in all pear chromosomes. Segmental duplication and tandem duplication were identified as the main patterns contributors to the expansion of *PRX* gene expansion in pear. In addition, a sliding-window analysis of Ka/Ks ratios showed some amino acid site under positive selection. Finally, expressed sequence tags analysis revealed that 41 *PbPRX* genes are expressed during fruit development. Furthermore, qRT-PCR analysis showed that five *PbPRX* genes from subgroup C might be involved in the regulation of lignin synthesis in pear fruit. This present study increases our understanding of *PRX* genes in pear, as well as lays the foundation for further clarify of the biological functions of these PRX proteins in other plants.

## Author contributions

YCao and YCai conceived and designed the experiments; YCao performed the experiments; YCao, YH, DM, DL, and QJ analyzed the data; YCao, YH, YL, and YCai contributed reagents/materials/analysis tools; YCao and YH wrote the paper.

### Conflict of interest statement

The authors declare that the research was conducted in the absence of any commercial or financial relationships that could be construed as a potential conflict of interest.

## Supplementary material

Figure S1 **Phylogenetic tree reconstruction of PRX proteins**. The PRX proteins from the pear, yang mei, peach and strawberry were aligned using Clustalw software, and the neighbor-joining tree was constructed by MEGA7.0 software, with1000 bootstrap replicates.

Figure S2 **Sliding window plots of duplicated *PRX* genes in pear**. The window size is 150 bp, and the step size is 9 bp. The positions of the PRX domain were represented by gray blocks, respectively.

Table S1 **Primers in this study**.

Table S2 **The detailed information of *PRX* genes in pear**.

Table S3 **All MEME motif sequences in pear PRX proteins**.

Table S4 **Orthologous analyses of PRX genes between pear, *Populus* and *Arabidopsis thaliana***.

Table S5 **Estimates of the divergence time for pear duplicated PRX paralogs**.

Table S6 **The ESTs of putative *PbPRX* genes**.
