# Supplementary figures and images for: Structural, Evolutionary, and Functional Analysis of the Class III Peroxidase Gene Family in Chinese Pear (Pyrus bretschneideri)
Source: Front Plant Sci. 2016 Dec 9;7:1874. doi: 10.3389/fpls.2016.01874 (PMC5145892; doi:10.3389/fpls.2016.01874)

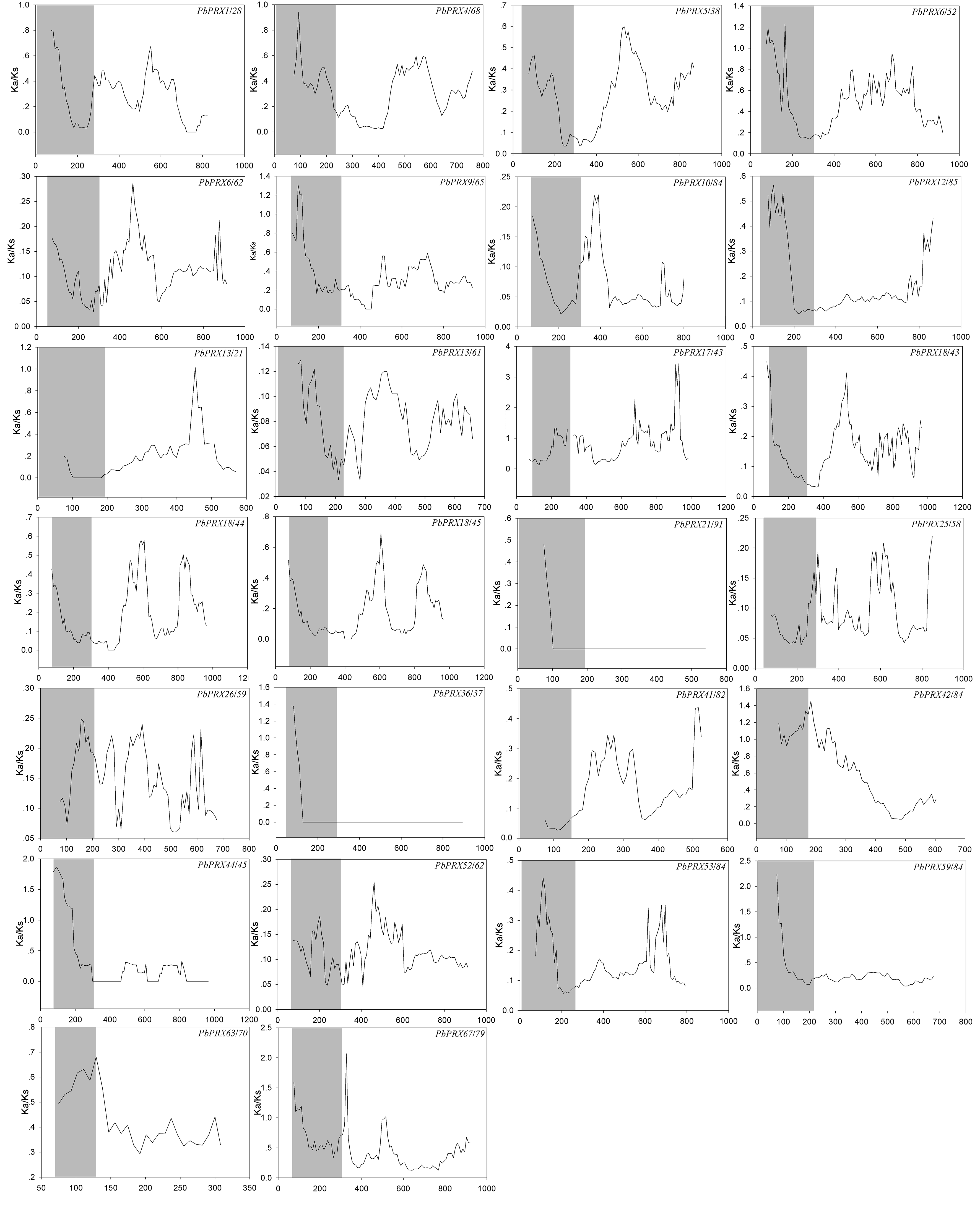

Supplement: Figure S2 — Sliding window plots of duplicated PRX genes in pear. The window size is 150 bp, and the step size is 9 bp. The positions of the PRX domain were represented by gray blocks, respectively. [file Image2.TIF]
